# Plasma leucine-rich α-2 glycoprotein 1 in ST-elevation myocardial infarction: vertical variation, correlation with T helper 17/regulatory T ratio, and predictive value on major adverse cardiovascular events

**DOI:** 10.3389/fcvm.2024.1326897

**Published:** 2024-04-29

**Authors:** Ting Luo, Xiaoli Jiang, Zhenzhen Zhang, Ming Gao, Hao Wang

**Affiliations:** ^1^ Department of Cardiology, The First People’s Hospital of Chengdu, Chengdu, China; ^2^ Department of Cardiology, The Central Hospital of Wuhan, Wuhan, China

**Keywords:** ST-elevation myocardial infarction, leucine-rich α-2 glycoprotein 1, T helper 17 cells, regulatory T cells, major adverse cardiovascular event

## Abstract

**Objective:**

Leucine-rich α-2 glycoprotein 1 (LRG1) promotes inflammation and myocardial injury, but its clinical role in ST-elevation myocardial infarction (STEMI) is rarely disclosed. Herein, this prospective study aimed to explore the value of plasma LRG1 at different time points to predict major adverse cardiovascular event (MACE) risk in patients with STEMI.

**Methods:**

In total, 209 patients with STEMI were enrolled for determining plasma LRG1 at admission and on day (D)1/D7/D30 after admission via enzyme-linked immunosorbent assay, as well as for determination of peripheral blood T helper 17 (Th17) cells and regulatory T (Treg) cells by flow cytometry. In addition, plasma LRG1 was obtained from 30 healthy controls at enrollment.

**Results:**

LRG1 was increased in patients with STEMI at admission compared with healthy controls (*P *< 0.001). In patients with STEMI, LRG1 varied at different time points (*P *< 0.001), which elevated from admission to D1, and gradually declined thereafter. LRG1 at admission was positively associated with Th17 cells (*P *= 0.001) and Th17/Treg ratio (*P *= 0.014). LRG1 at admission (*P *= 0.013), D1 (*P *= 0.034), D7 (*P *= 0.001), and D30 (*P *= 0.010) were increased in patients with MACE compared with those without. LRG1 at D7 exhibited good ability to estimate MACE risk (area under curve = 0.750, 95% confidence interval = 0.641–0.858). LRG1 at admission > 60 μg/ml (*P *= 0.031) and D7 > 60 μg/ml (*P *= 0.018) were linked with increased accumulating MACE. Importantly, LRG1 at D7 > 60 μg/ml was independently correlated with increased MACE risk (hazard ratio = 5.216, *P *= 0.033).

**Conclusion:**

Plasma LRG1 increases from admission to D1 and gradually declines until D30, which positively links with Th17 cells and MACE risk in patients with STEMI.

## 1 Introduction

ST-elevation myocardial infarction (STEMI) is a common manifestation of acute coronary syndrome, the incidence of which increased from 1990 to 2015 in China (1, 2). It is typically caused by thrombotic occlusion at the location of ruptured atherosclerotic plaques, resulting in cardiac hypoxia and ischemia (3). Timely reperfusion therapy, including percutaneous coronary intervention (PCI) and secondary prevention (such as fibrinolytic therapy and coronary artery bypass surgery), reduces the mortality of patients with STEMI (4, 5). Regrettably, some patients with STEMI have major adverse cardiovascular events (MACEs) following reperfusion treatment, remaining a challenge for the clinical management of STEMI (6–8). Therefore, identifying some biomarkers with the potential to estimate MACE risk early can assist in providing timely and personalized intervention for patients with STEMI.

Leucine-rich α-2 glycoprotein 1 (LRG1), mainly produced by hepatocytes, is a secretory glycoprotein composed of eight leucine-rich repeated sequences, and each of the sequences consists of 24 amino acid residues (9, 10). As an essential upstream of the transforming growth factor-β (TGF-β) signaling, LRG1 promotes pathological angiogenesis, inflammation, and T helper (Th) 17 cell differentiation, which further facilitate the progression of atherosclerosis (11–13). In addition, LRG1 also facilitates hypoxia-induced cardiomyocyte apoptosis and autophagy by regulating hypoxia-inducible factor-1α (HIF-1α) (14). Given these biological roles of LRG1, recent studies have investigated the ability of LRG1 to predict the occurrence of cardiovascular events in high-risk populations, such as patients with end-stage renal disease and diabetes (15–18). For example, a study discloses that increased plasma LRG1 is linked with a higher risk of cardiovascular disease in patients with end-stage renal disease (15). Another study identifies that elevated LRG1 is independently linked with increased occurrence of heart failure in type 2 diabetes patients (17). Nonetheless, the ability of LRG1 in estimating the MACE of patients with STEMI has not been explored.

Hence, this prospective study quantified plasma LRG1 at different time points in 209 patients with STEMI, aiming to investigate its linkage with Th17 cells, regulatory T (Treg) cells, and MACE risk in these patients.

## 2 Methods

### 2.1 Patients and healthy controls

This study enrolled 209 patients with STEMI who were treated between April 2020 and September 2022. The inclusion criteria were as follows: (a) the patients had a diagnosis of STEMI, (b) were aged ≥18 years, (c) received PCI treatment after admission, and (d) were willing to cooperate during follow-up. The exclusion criteria were as follows: (a) The patient had a serious infection, (b) had an organ transplant, and (c) had cancer or malignant hematologic disease. A total of 30 healthy subjects were enrolled as controls. The inclusion criteria were as follows: (a) The patient had a normal physical examination before enrollment and (b) were aged ≥18 years. The exclusion criteria were referred to those of the patients with STEMI. The study was approved by the Ethics Committee of the First People's Hospital of Chengdu. Every patient and healthy subject signed a written informed consent form.

### 2.2 Data collection, sample collection, and sample testing

For the patients with STEMI, baseline characteristics were collected after the admission of the patients. Peripheral blood was collected from patients at admission, day 1 (D1), day 7 (D7), and day 30 (D30) after admission. Peripheral blood was also collected from healthy subjects after enrollment.

A portion of peripheral blood from patients (collected at admission, D1, D7, and D30 after admission), as well as from healthy subjects (collected after the enrollment), was centrifuged to obtain plasma, which was used to detect LRG1 by the enzyme-linked immunosorbent assay (ELISA). An ELISA kit (catalog no: E-EL-H6067; Elabscience, China) was used. Another portion of peripheral blood from the patients (collected at admission or D1 after admission) was used to detect Th17 cells and Treg cells by flow cytometry (FCM). A Th17/Treg Phenotyping Kit (catalog no: AB_2869366, BD, USA) was used.

### 2.3 Follow-up

The follow-up period of patients with STEMI lasted until November 2022, with a median of 14.9 months and a range of 1.0–29.3 months. MACE (death, myocardial infarction, or revascularization) (19) and the time of occurrence during the follow-up were recorded.

### 2.4 Data analysis

In this study, the data were analyzed using the software SPSS 25.0 (IBM Corp., USA). The patients who died within 24 h after admission were excluded from the data analysis. The Mann–Whitney *U* test was used for comparisons between two groups (LRG1 comparison between healthy control and patients at admission; LRG1 comparison between patients who presented with MACE and did not present with MACE at admission, D1, D7, and D30 after admission), and the Friedman test was used for comparisons among three or more matched groups (LRG1 comparison among patients at admission, D1, D7, and D30 after admission). The Spearman test was used for the correlation analysis (between LRG1 at admission and Th17, Treg cells, and Th17/Treg ratio). The receiver operating characteristic (ROC) analyses demonstrated the predictive efficacy of LRG1 at different times (at admission, D1, D7, and D30 after admission) for MACE. Kaplan–Meier (KM) curves presented the relationship between LRG1 (cut-off value was 60 μg/ml) at different times (at admission, D1, D7, and D30 after admission) and MACE. The log-rank test was the method of analysis. Univariate and step-forward multivariate Cox regression analyses demonstrated independent factors that were associated with MACE, presented by forest plot. *P*-values less than or equal to 0.05 were considered to indicate statistical significance.

## 3 Results

### 3.1 Clinical characteristics of patients with STEMI

The median (range) age of the 209 STEMI patients was 61.0 (40.0–83.0) years, and 80 (38.3%) patients were ≥65 years. In addition, 154 (73.7%) patients were male. A total of 147 (70.3%), 93 (44.5%), and 56 (26.8%) patients had a history of hypertension, hyperlipidemia, and diabetes mellitus, respectively. The median [interquartile range (IQR)] symptom-to-balloon time was 180.0 (135.0–250.0) min, and this time in 168 (80.4%) patients was ≥130 min. Moreover, 79 (37.8%) patients had multivessel disease. The median (IQR) infarct size was 24.0% (18.0%–29.0%). The detailed clinical information is displayed in Table 1. Furthermore, the median (IQR) Th17 cells, Treg cells, and Th17/Treg ratio were 4.4% (2.4%–6.1%), 6.4% (4.8%–7.8%), and 0.6% (0.4%–1.1%), correspondingly. The levels of biochemical indicators in patients with STEMI are provided in Table 2.

**Table 1:** Characteristics of patients with STEMI.

Variables	STEMI patients (*N* = 209)
Age (years)	
Median (range)	61.0 (40.0–83.0)
≥65 years, *n* (%)	80 (38.3)
Male, *n* (%)	154 (73.7)
Body mass index (kg/m^2^)	
Mean ± SD	24.9 ± 3.4
≥28 kg/m^2^, *n* (%)	41 (19.6)
Current smoker, *n* (%)	83 (39.7)
History of hypertension, *n* (%)	147 (70.3)
History of hyperlipidemia, *n* (%)	93 (44.5)
History of diabetes mellitus, *n* (%)	56 (26.8)
Symptom-to-balloon time (min)	
Median (IQR)	180.0 (135.0–250.0)
≥130 min, *n* (%)	168 (80.4)
Culprit lesion, *n* (%)	
Left anterior descending artery	83 (39.7)
Left circumflex artery	44 (21.1)
Right coronary artery	82 (39.2)
Multivessel disease, *n* (%)	79 (37.8)
Thrombus aspiration, *n* (%)	47 (22.5)
Number of implanted stents, *n* (%)	
1	167 (79.9)
2	42 (20.1)
Type of stent, *n* (%)	
Sirolimus-eluting stent	135 (64.6)
Everolimus-eluting stent	74 (35.4)
Stent diameter (mm)	
Median (IQR)	3.0 (3.0–3.5)
≥3.0 mm^a^, *n* (%)	166 (79.4)
Stent length (total) (mm)	
Median (IQR)	33.0 (23.0–38.0)
≥33 mm^a^, *n* (%)	111 (53.1)
Infarct size (%)	
Median (IQR)	24.0 (18.0–29.0)
≥24%^a^, *n* (%)	101 (48.3)

SD, standard deviation.

^a^ The selected cut-off values were based on the median values.

**Table 2:** Biochemical indicators and CD4^+^ T cells of patients with STEMI.

Variables	STEMI patients (*N* = 209)
WBC (10^9^/L)	
Median (IQR)	10.1 (7.8–12.8)
≥10 × 10^9^/L^a^, *n* (%)	103 (49.3)
FBG (mmol/L)	
Median (IQR)	5.2 (4.4–6.3)
≥6.2 mmol/L^a^, *n* (%)	59 (28.2)
Scr (μmol/L)	
Median (IQR)	84.1 (73.8–98.7)
≥110 μmol/L^a^, *n* (%)	27 (12.9)
TG (mmol/L)	
Median (IQR)	1.7 (1.0–2.4)
≥1.7 mmol/L^a^, *n* (%)	113 (54.1)
TC (mmol/L)	
Median (IQR)	4.5 (3.8–5.5)
≥5.2 mmol/L^a^, *n* (%)	61 (29.2)
LDL-C (mmol/L)	
Median (IQR)	3.1 (2.3–3.9)
≥3.4 mmol/L^a^, *n* (%)	84 (40.2)
HDL-C (mmol/L)	
Median (IQR)	1.0 (0.9–1.2)
≤0.94 mmol/L^a^, *n* (%)	78 (37.3)
CRP (mg/L)	
Median (IQR)	4.9 (3.3–6.8)
≥5 mg/L^a^, *n* (%)	95 (45.5)
cTnI (ng/ml)	
Median (IQR)	4.4 (3.0–6.5)
≥4.4 ng/ml^a^, *n* (%)	107 (51.2)
CK-MB (ng/ml)	
Median (IQR)	36.7 (18.8–56.0)
≥32.5 ng/ml^a^, *n* (%)	116 (55.5)
Th17 cells (%)	
Median (IQR)	4.4 (2.4–6.1)
≥4.4%^b^, *n* (%)	81 (50.0)
Treg cells (%)	
Median (IQR)	6.4 (4.8–7.8)
≥6.4%^b^, *n* (%)	81 (50.0)
Th17/Treg ratio	
Median (IQR)	0.6 (0.4–1.1)
≥0.6^b^, *n* (%)	90 (55.6)

FBG, fasting plasma glucose; Scr, serum creatinine; TG, triglyceride; TC, total cholesterol; LDL-C, low-density lipoprotein cholesterol; HDL-C, high-density lipoprotein cholesterol; CK-MB, creatinine kinase-myocardial band.

^a^ The selected cut-off values were based on the normal range of clinical indicators.

^b^ The selected cut-off values were based on the median values.

### 3.2 Plasma LRG1 in patients with STEMI at different time points and in healthy controls

**Figure 1:**
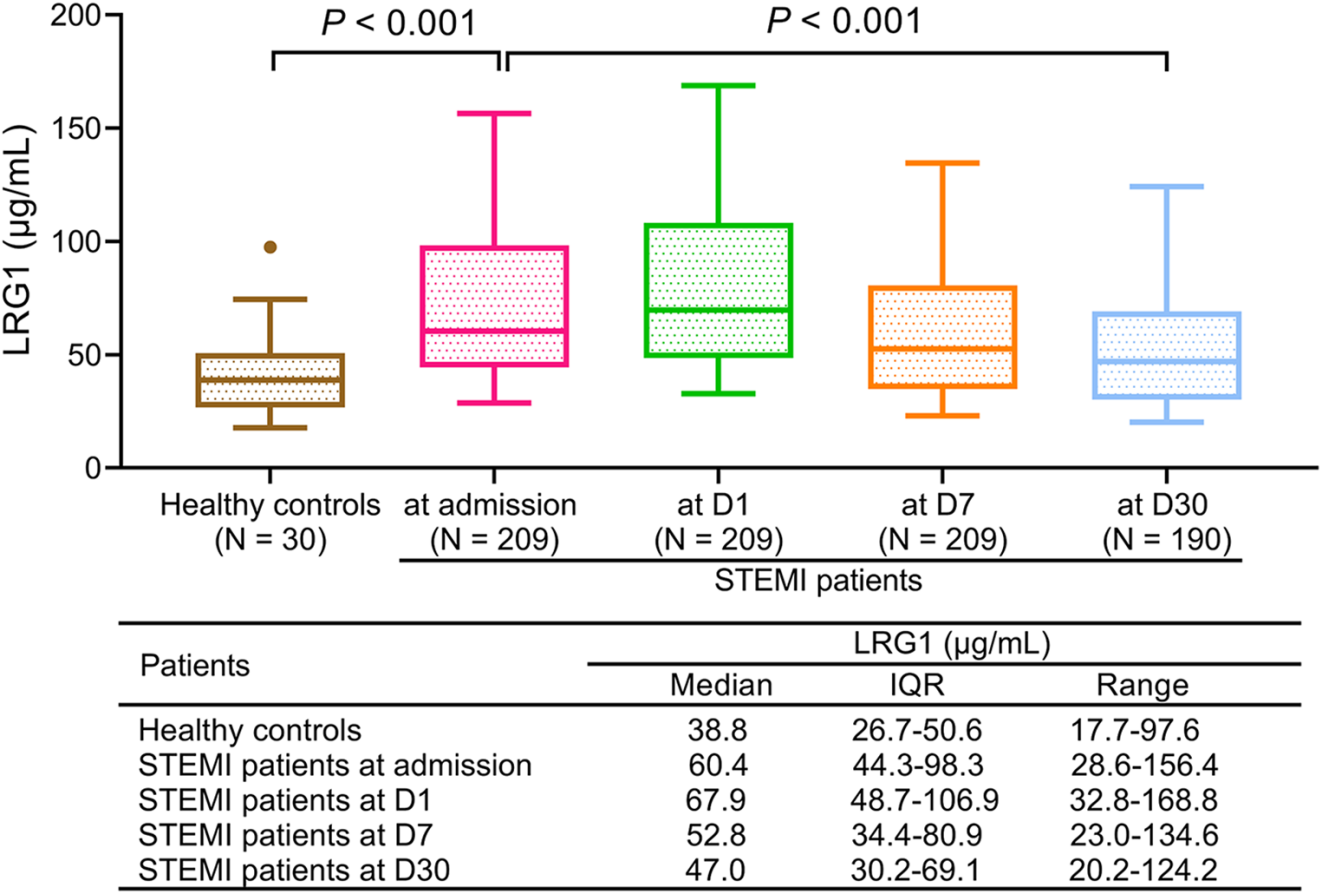
Plasma LRG1 in patients with STEMI was elevated compared with in healthy controls and varied at different time points.

Plasma LRG1 was increased in patients with STEMI at admission compared with healthy controls [median (IQR): 60.4 (44.3–98.3) μg/ml vs. 38.8 (26.7–50.6) μg/ml, *P *< 0.001]. In patients with STEMI, plasma LRG1 at different time points was varied (*P *< 0.001), which elevated from admission to D1, and gradually declined thereafter. Furthermore, the median (IQR) plasma LRG1 at D1, D7, and D30 were 67.9 (48.7–106.9), 52.8 (34.4–80.9), and 47.0 (30.2–69.1) μg/ml, respectively (Figure 1).

### 3.3 Correlation of plasma LRG1 with clinical characteristics of patients with STEMI

Plasma LRG1 at admission was not linked with a history of hypertension (*P *= 0.412) or diabetes mellitus (*P *= 0.585), while plasma LRG1 at admission was negatively related to a history of hyperlipidemia (*P *= 0.017) in patients with STEMI (Supplementary Figures S1A–C). Plasma LRG1 at admission positively correlated with cardiac troponin (cTnI) (*P *= 0.002) (Supplementary Figure S1D) and C-reactive protein (CRP) (*P *< 0.001) (Supplementary Figure S1E). In addition, plasma LRG1 at admission was positively related to white blood count (WBC) (*P *= 0.001), but not infarct size (*P *= 0.232) or symptom-to-balloon time (*P *= 0.224), in patients with STEMI (Supplementary Figures S1F–H).

### 3.4 Correlation of plasma LRG1 with Th17 cells and Treg cells in patients with STEMI

**Figure 2:**
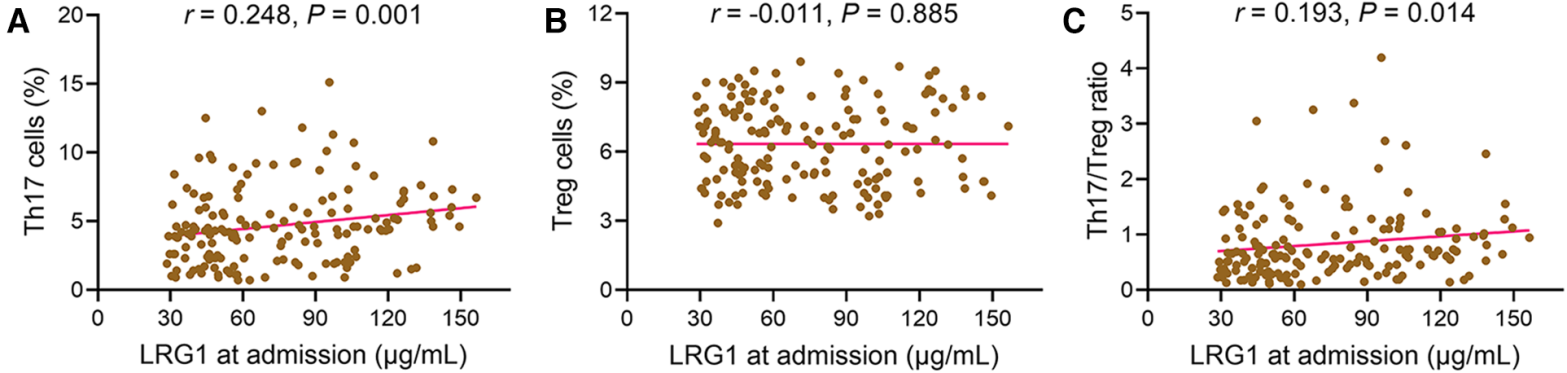
Plasma LRG1 at admission was positively related to Th17 cells and Th17/Treg ratio in patients with STEMI. Associations of plasma LRG1 at admission with (**A**) Th17 cells, (**B**) Treg cells, and (**C**) Th17/Treg ratio in patients with STEMI.

Plasma LRG1 at admission was positively associated with Th17 cells in patients with STEMI (*r *= 0.248, *P *= 0.001) (Figure 2A). However, plasma LRG1 at admission was not related to Treg cells (*r *= −0.011, *P *= 0.885) (Figure 2B). In addition, increased plasma LRG1 at admission was linked with elevated Th17/Treg ratio (*r *= 0.193, *P *= 0.014) (Figure 2C).

### 3.5 Comparison of plasma LRG1 at different time points between patients with STEMI with and without MACE

**Figure 3:**
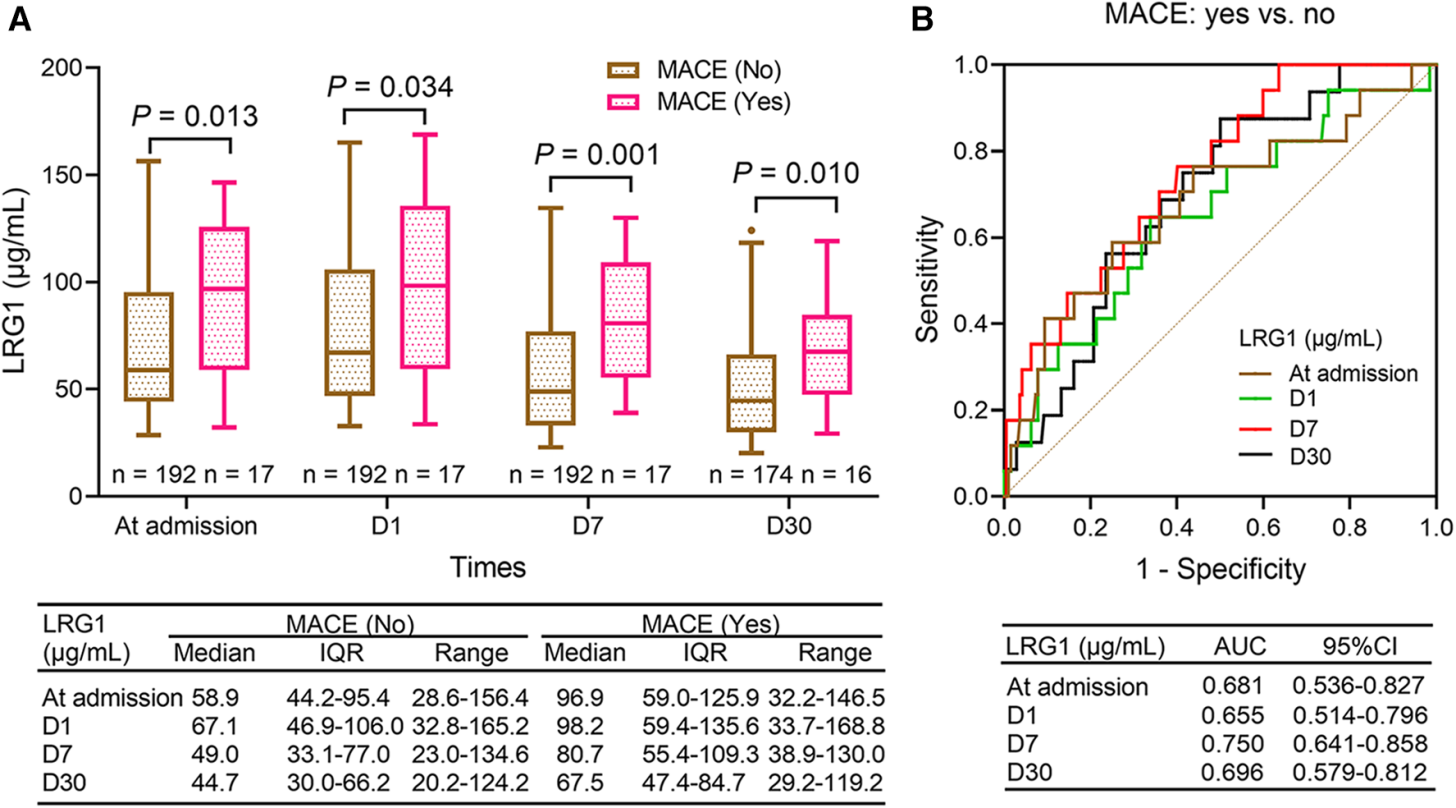
Plasma LRG1 at admission, D1, D7, and D30 were elevated in patients with STEMI with MACE occurrence compared with those without MACE occurrence. (**A**) Comparison of plasma LRG1 at admission, D1, D7, and D30 between patients with STEMI with and without MACE (**B**) ROC curves reflecting the ability of plasma LRG1 at admission, D1, D7, and D30 to estimate MACE risk in patients with STEMI.

Plasma LRG1 at admission (*P *= 0.013), D1 (*P *= 0.034), D7 (*P *= 0.001), and D30 (*P *= 0.010) were increased in patients with STEMI with MACE compared with those without (Figure 3A). Plasma LRG1 at D7 exhibited a good ability to estimate MACE risk in patients with STEMI [area under the curve (AUC): 0.750, 95% confidence interval (CI): 0.641–0.858], whereas plasma LRG1 at admission (AUC: 0.681, 95% CI: 0.536–0.827), D1 (AUC: 0.655, 95% CI: 0.514–0.796), and D30 (AUC: 0.696, 95% CI: 0.579–0.812) showed a general predictive value for MACE risk (Figure 3B).

### 3.6 Association of plasma LRG1 at different time points with accumulating MACE in patients with STEMI

**Figure 4:**
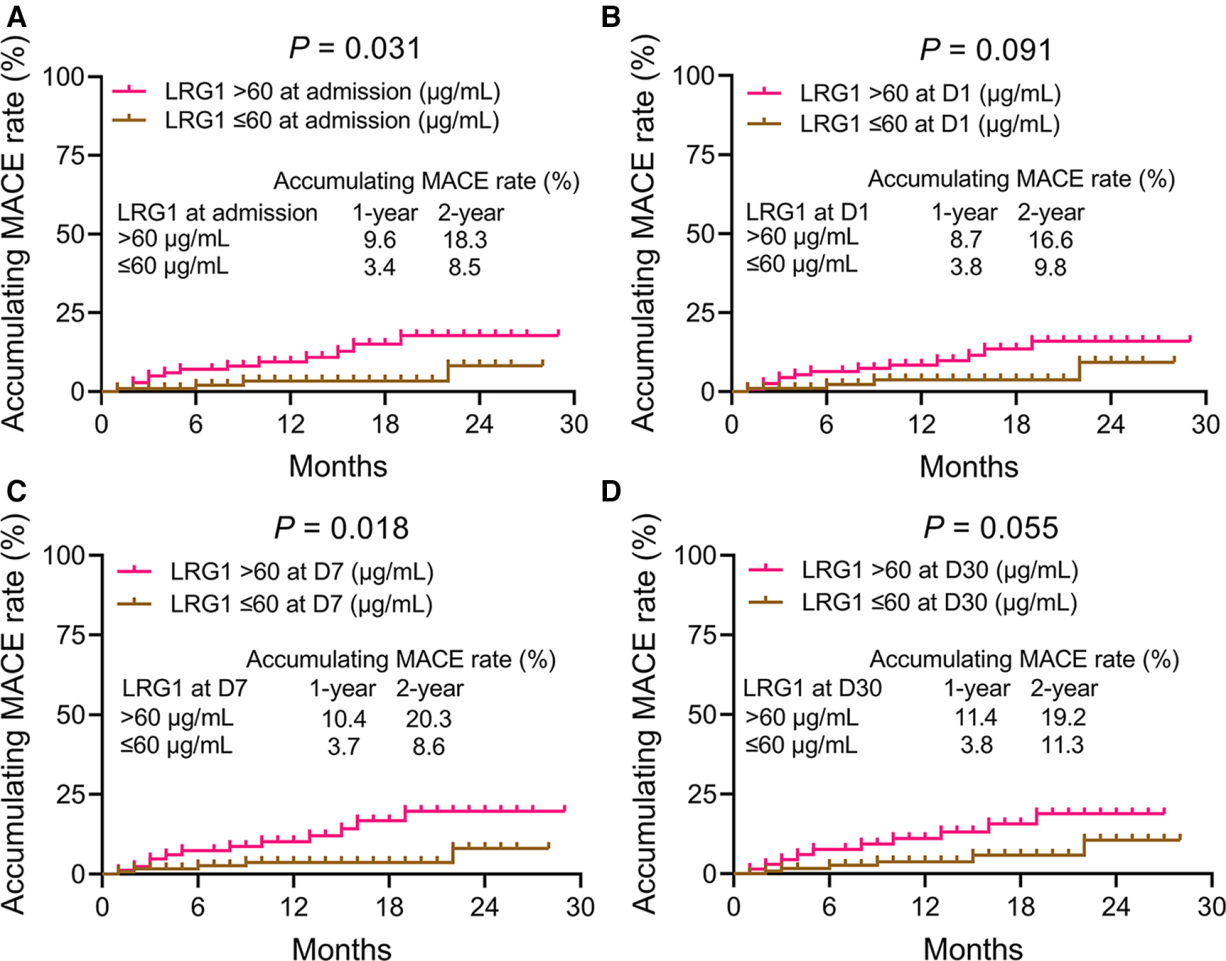
Accumulating MACE was elevated in patients with STEMI with plasma LRG1 at admission and at D7 >60 vs. ≤60 μg/ml. Comparison of accumulating MACE between patients with STEMI with plasma LRG1 at admission >60 and ≤60 μg/ml (**A**) Comparison of accumulating MACE between patients with STEMI with plasma LRG1 at D1 >60 and ≤60 μg/ml (**B**) Comparison of accumulating MACE between patients with STEMI with plasma LRG1 at D7 >60 and ≤60 μg/ml (**C**) Comparison of accumulating MACE between patients with STEMI with plasma LRG1 at D30 >60 and ≤60 μg/ml (**D**).

Plasma LRG1 at admission >60 μg/ml was related to elevated MACE in patients with STEMI (*P *= 0.031). The 1-year and 2-year accumulative MACE rates were 9.6% and 18.3% in patients with plasma LRG1 at admission >60 μg/ml, while the rates were 3.4% and 8.5% in those with plasma LRG1 at admission ≤60 μg/ml, respectively (Figure 4A). Plasma LRG1 at D1 > 60 μg/ml was not linked with MACE (*P *= 0.091) (Figure 4B). Plasma LRG1 at D7 > 60 μg/ml was correlated with increased MACE (*P *= 0.018). Specifically, the 1-year and 2-year accumulating MACE rates were 10.4% and 20.3%, respectively, in patients with plasma LRG1 at D7 > 60 μg/ml, but they were 3.7% and 8.6%, respectively, in patients with plasma LRG1 at D7 ≤ 60 μg/ml (Figure 4C). Differently, plasma LRG1 at D30 > 60 μg/ml was not related to MACE (*P *= 0.055) (Figure 4D).

### 3.7 Factors affecting MACE in patients with STEMI

**Figure 5:**
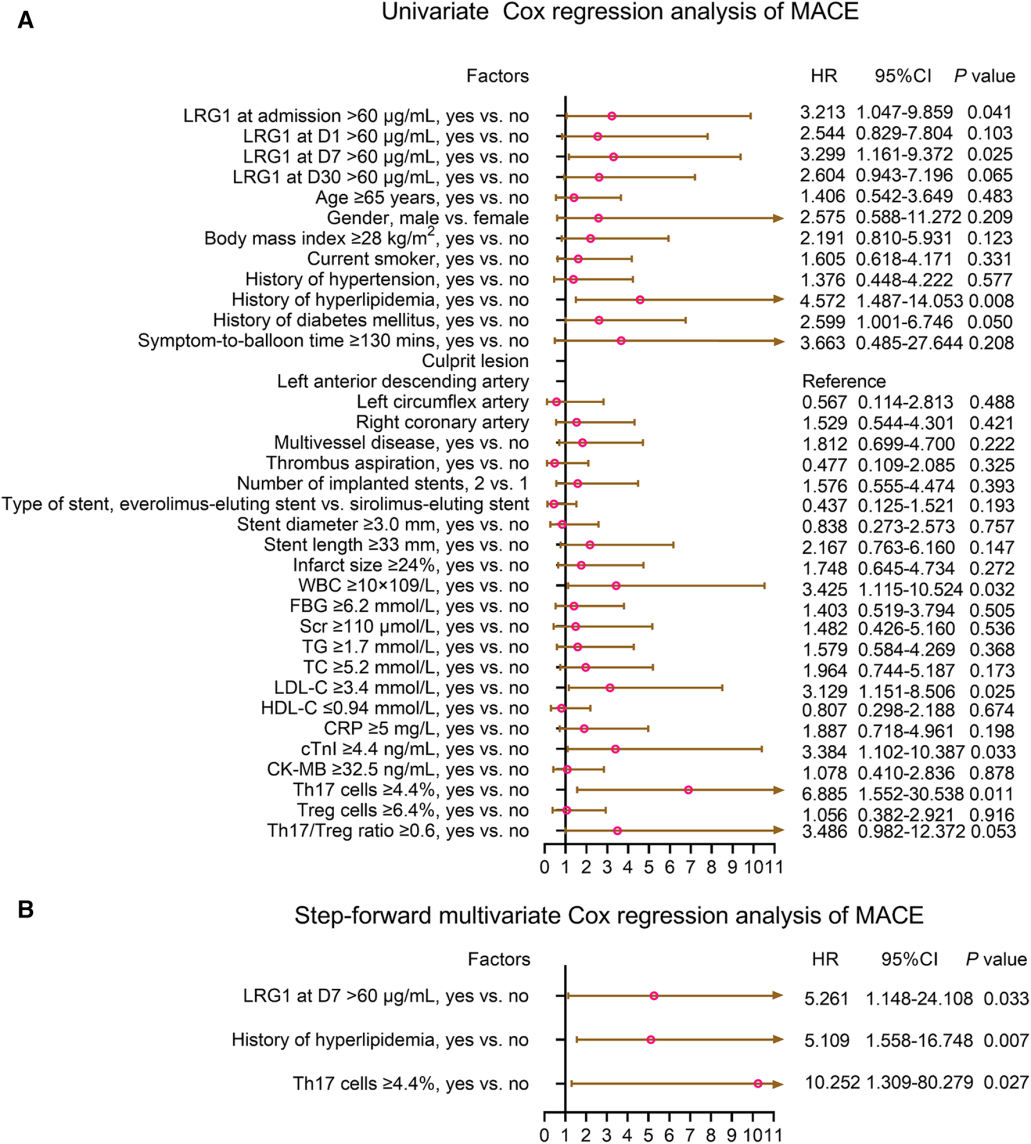
Plasma LRG1 at D7 >60 μg/ml (yes vs. no) was independently associated with increased MACE risk in patients with STEMI. (**A**) Univariate and (**B**) step-forward multivariate Cox regression analysis of MACE in patients with STEMI.

Plasma LRG1 at admission >60 μg/ml (yes vs. no) [hazard ratio (HR) = 3.213, *P *= 0.041], plasma LRG1 at D7 > 60 μg/ml (yes vs. no) (HR = 3.299, *P *= 0.025), history of hyperlipidemia (yes vs. no) (HR = 4.572, *P *= 0.008), history of diabetes mellitus (yes vs. no) (HR = 2.599, *P *= 0.050), WBC ≥10 × 10^9^/L (yes vs. no) (HR = 3.425, *P *= 0.032), LDL-C ≥ 3.4 mmol/L (yes vs. no) (HR = 3.129, *P *= 0.025), cTnI ≥4.4 ng/ml (yes vs. no) (HR = 3.384, *P *= 0.033), and Th17 cells ≥4.4% (yes vs. no) (HR = 6.885, *P *= 0.011) were related to elevated MACE risk. After adjustment by the step-forward multivariate Cox regression, plasma LRG1 at D7 > 60 μg/ml (yes vs. no) (HR = 5.216, *P *= 0.033), history of hyperlipidemia (yes vs. no) (HR = 5.109, *P *= 0.007), and Th17 cells ≥4.4% (yes vs. no) (HR = 10.252, *P *= 0.027) were independently associated with increased MACE risk (Figure 5).

## 4 Discussion

Several bioinformatic analyses have shown that LRG1 may serve as a useful biomarker in cardiovascular diseases (20–23). For instance, one study demonstrated that LRG1 is elevated in patients with acute coronary syndrome compared with healthy controls (20). Another proteomic analysis revealed that LRG1 may be a potential biomarker for early diagnosis of heart failure (22). The current study noticed that plasma LRG1 was increased in patients with STEMI than in healthy controls. Moreover, plasma LRG1 was elevated from admission to D1, and then gradually decreased until D30 in patients with STEMI. The possible explanations might be as follows: (1) LRG1, as a vasculopathy factor, is positively associated with abnormal angiogenesis and acute inflammation; meanwhile, the latter factors are enhanced in patients with STEMI (11, 20). Hence, plasma LRG1 was increased in patients with STEMI compared with healthy controls. (2) Reperfusion therapy might induce acute inflammation in patients with STEMI at first, and the inflammation level gradually declines following the coronary artery recanalization (24); furthermore, LRG1 was positively correlated with inflammation level according to the previous reports (16, 25). Therefore, LRG1 initially increased but then gradually decreased.

The level of LRG1 in hypertension/hyperlipidemia is rarely reported. For hypertension, only one study discloses that serum LRG1 ranges from 11.3 to 28.1 μg/ml in patients with asymptomatic hypertension with high B-type natriuretic peptide, and it ranges from 9.5 to 17.0 μg/ml in those with low B-type natriuretic peptide (22). For hyperlipidemia, only one previous study shows that LRG1 is a potential protein biomarker for coronary atherosclerosis in strain-treated patients with familial hypercholesterolemia, but its detailed level is not mentioned in the previous study (26). For diabetes, several studies have revealed that elevated LRG1 is associated with renal dysfunction in patients with diabetes mellitus (27–29). Subsequently, it is assumed that plasma LRG1 is elevated in patients with hypertension, hyperlipidemia, and diabetes before developing into STEMI compared with healthy subjects. Regrettably, the current study only enrolled healthy subjects as controls, but it did not include patients with hypertension, hyperlipidemia, and diabetes. Hence, this issue remains for further investigation.

Th17 cells and Treg cells, as critical effector T cells in adaptive immunity, promote inflammation during cardiac tissue remodeling and lead to the pathogenesis of cardiac fibrosis (30, 31). Moreover, it has been previously reported that LRG1 favors immune cell participation in systemic inflammation, which enhances the differentiation of naïve CD4^+^ T cells into pro-inflammatory Th17 lymphocytes by elevating interleukin 6 receptor; however, LRG1 has no influence on Treg differentiation (13). Furthermore, clinical evidence of the correlation of LRG1 with Th17 cells and Treg cells is lacking in patients with STEMI. The current study identified that plasma LRG1 was positively correlated with Th17 cells and Th17/Treg ratio in patients with STEMI, but it was not related to Treg cells, which showed a similar trend to the *in vitro* study (13).

In terms of the prognosis, this study revealed that patients with STEMI with MACE had increased plasma LRG1 at admission, D1, D7, and D30 compared with those without. Moreover, both ROC curves and KM curves displayed the good predictive value of plasma LRG1 on MACE risk. More importantly, plasma LRG1 at D7 > 60 μg/ml was an independent risk factor for MACE in patients with STEMI, whose correlation with MACE was not affected by any other factor (including other independent factors and other factors included in the multivariate Cox regression model). Thus, LRG1 stayed predictive for MACE after compensation for hyperlipidemia, diabetes, cTnI, and CRP in patients with STEMI. The probable reasons for these findings are as follows: (1) LRG1 induces a series of biological processes, including inflammatory response, Th17 differentiation, and endothelial dysfunction, to facilitate atherosclerotic progression and increase the risk of plaque rupture, which further results in a worse prognosis in patients with STEMI (13, 32). (2) LRG1 exacerbates myocardial cell apoptosis and cardiac fibrosis, which influences cardiac function (14, 33, 34). Combining the aforementioned two aspects, plasma LRG1 was therefore a potential biomarker for predicting MACE in patients with STEMI. On the contrary, it is reported that LRG1 is an inhibitor for adverse cardiac remodeling, and its deficiency aggravates myocardial fibrosis with cardiac dysfunction after myocardial infarction (35, 36). Considering that adverse cardiac remodeling is also a risk factor for MACE occurrence (37), it seems that LRG1 may own both protective and adverse roles in STEMI outcomes, which requires more investigations.

Some inevitable limitations of this study were as follows. First, LRG1 was quantified within 30 days after admission, but its variation in the long term was unclear. Second, this study only evaluated the clinical role of LRG1 in patients with STEMI, while it lacked investigation into other types of cardiovascular diseases (such as non-STEMI and angina pectoris). Third, the correlation of LRG1 with other CD4^+^ T cells, such as Th1, Th2, Th9, and Th22 cells, was unknown in the present study. Fourth, all patients enrolled in this study were from China, and this issue limited the generalization of the findings, which requires validation in multiple regions in the future.

Collectively, plasma LRG1 elevates from admission to D1 and then gradually declines, and its elevated level correlates with increased Th17 cells and MACE risk in patients with STEMI. In addition, plasma LRG1 at D7 > 60 μg/ml possesses good predictive value for MACE in these patients.

## Data Availability

The original contributions presented in the study are included in the article/**Supplementary Material**, further inquiries can be directed to the corresponding author.

## Supplementary material

Supplementary Figure S1

Plasma LRG1 at admission negatively linked with history of hyperlipidemia, but positively related to cTnI, CRP, and WBC in STEMI patients. Association of plasma LRG1 at admission with history of hypertension (**A**), history of hyperlipidemia (**B**), history of diabetes mellitus (**C**), cTnI (**D**), CRP (**E**), infarct size (**F**), WBC (**G**), and symptom-to-balloon time (H) in STEMI patients.
