# Supplementary figures and images for: Plasma leucine-rich α-2 glycoprotein 1 in ST-elevation myocardial infarction: vertical variation, correlation with T helper 17/regulatory T ratio, and predictive value on major adverse cardiovascular events
Source: Front Cardiovasc Med. 2024 Apr 29;11:1326897. doi: 10.3389/fcvm.2024.1326897 (PMC11089199; doi:10.3389/fcvm.2024.1326897)

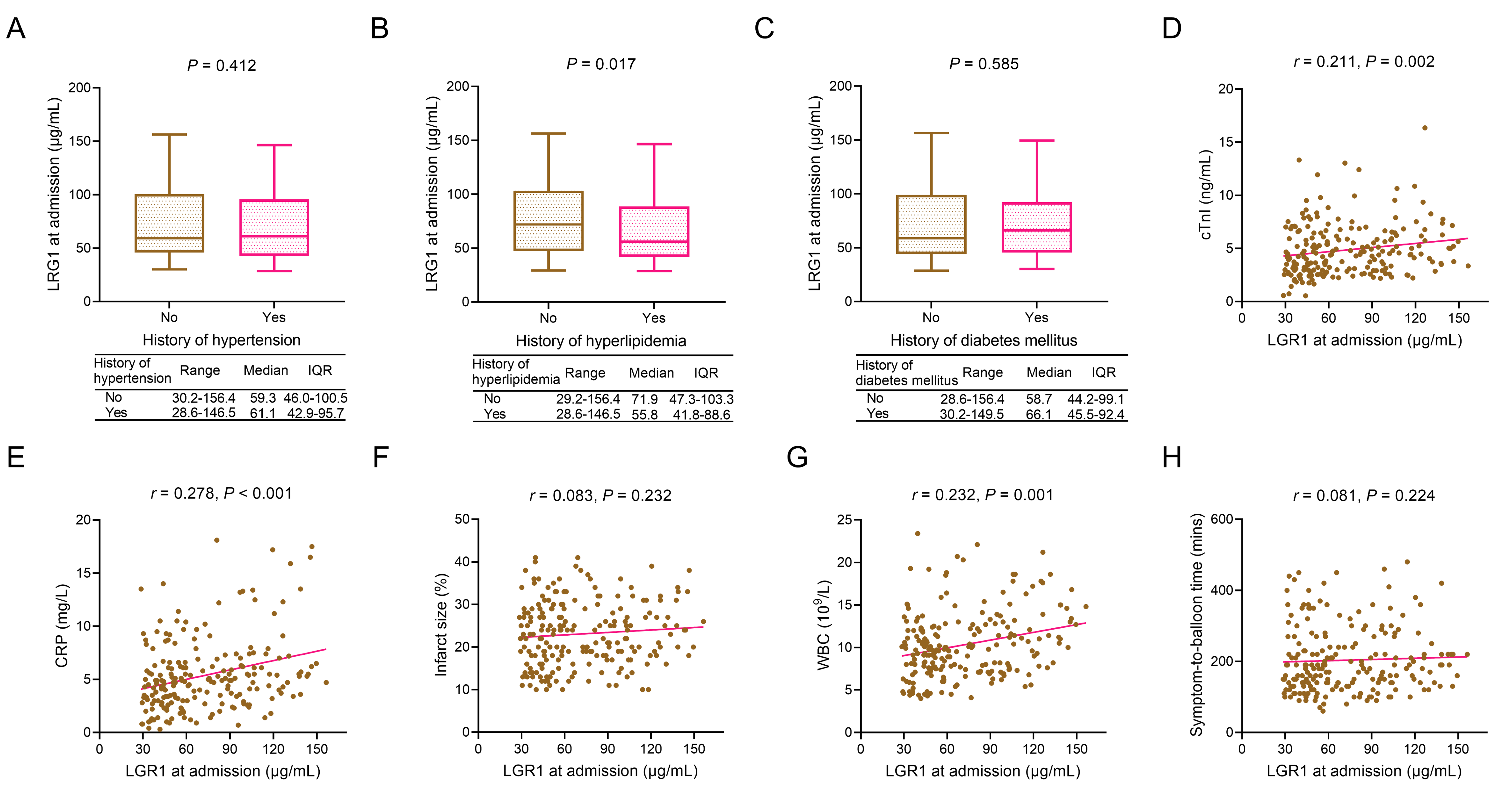

Supplement: Supplementary file 1 [file Image1.tif]
